# Association of Psychiatric Disorders with Pedestrian Safety Behaviors: Baseline Findings from Persian Traffic Cohort

**DOI:** 10.34172/aim.29961

**Published:** 2025-01-01

**Authors:** Mostafa Farahbakhsh, Fatemeh Niknami, Homayoun Sadeghi-Bazargani, Sanaz Noruzi, Sepideh Harzand-Jadidi

**Affiliations:** ^1^Research Centre of Psychiatry and Behavioural Sciences, Tabriz University of Medical Sciences, Tabriz, Iran; ^2^Road Traffic Injury Research Center, Tabriz University of Medical Sciences, Tabriz, Iran; ^3^Department of Psychiatry, School of Medicine, Tabriz University of Medical Sciences, Tabriz, Iran

**Keywords:** Anxiety disorder, Depressive disorder, Mental health, Pedestrian, Traffic accident, Traffic behavior

## Abstract

**Background:**

Mental health problems can disrupt traffic behaviors through reduced cognitive function, poor decision-making, increased behavioral errors, and concentration problems. This study aimed to examine the role of psychiatric disorders in pedestrians ‘ traffic behavior.

**Methods:**

This cross-sectional study was conducted on 275 pedestrians of the Persian Traffic Cohort (PTC) in 2022. The Pedestrian Traffic Behavior Questionnaire, Kessler’s Psychological Distress Scale, and the Structured Assessment of Personality Abbreviated Scale (SAPAS) screening questionnaires for people over 14 years were completed. Then, those who had a score above 3 on the SAPAS or a score above 20 on Kessler’s scale took part in a psychiatric interview by a psychiatrist or psychiatric resident. The data were analyzed in SPSS v. 26 via independent samples *t*-test, analysis of variance, and multiple linear regression.

**Results:**

The score of positive behaviors was significantly higher in pedestrians without depressive disorder than in those with depressive disorder (95% CI: 42.49–51.17, *P* value<0.001). The score of aggressive behaviors was significantly higher in pedestrians with depressive disorder than in those without (95% CI: 24.98–28.87, *P* value=0.001). There was no statistically significant difference between the two groups of pedestrians with and without generalized anxiety disorder in the scores of traffic behavior dimensions (*P* value>0.05).

**Conclusion:**

There was a significant relationship between pedestrians with depressive disorder and the score of positive and aggressive traffic behaviors. Meanwhile, the pedestrians’ anxiety disorder was not significantly related to any of their behavioral dimensions. More comprehensive studies should be conducted, taking into account more mental disorders and larger samples, to more precisely explain the impact of psychiatric disorders on pedestrians’ traffic behavior.

## Introduction

 Globally, pedestrian accidents account for a significant proportion of car accidents, with about 22% of the world’s traffic accidents involving pedestrians.^1,2^ According to studies conducted in Iran, more than 17 000 people die every year due to traffic accidents, 46% of whom are pedestrians.^3,4^ Therefore, pedestrians are vulnerable groups in traffic accidents. Their vulnerability in traffic accidents can be caused by various factors, including their age, sex, socioeconomic status, health status, and risky behaviors while crossing the street.^5,6^ Mental health problems are among the factors that can disrupt various aspects of life,^7^ especially one’s traffic behaviors, through reduced cognitive function, poor decision-making, increased behavioral errors, and concentration problems.^8-10^

 Mental health problems are common worldwide. Based on estimates, about 14% of the global burden of diseases is related to mental disorders.^11,12^ Depression and anxiety are the most common mental disorders today and impact many people as a global health problem. According to the literature, there is a significant relationship between mental disorders and traffic accidents, with anxiety and depression increasing the probability of traffic accidents by 2.5 times.^13^ According to Rahimi Movaghar, mood disorders such as depression can lead to high-risk driving. In cases of severe depression, high-risk driving can also raise the probability of traffic accidents.^14^ Another study reported a significant difference between average scores of paranoid ideation, obsessive-compulsive disorder, and depression in two groups of drivers who had or did not have accidents.^15^ According to Elander et al, there is a significant relationship between depression and accidents leading to injury.^16^ Soufi et al also reported a statistically significant correlation between motorcyclists’ driving behavior scores and their mental health. As a result, drivers are recommended to undergo careful psychological assessments when they apply for a driver’s license and even afterward to reduce the incidence of accidents.^17^

 In general, human factors in traffic accidents are divided into two categories: (1) factors that play a direct role at the moment of accidents (e.g. ignoring driving regulations, speeding, driving while sleepy and tired, drug and alcohol use); and (2) cognitive and psychological factors.^18^ Mental health experts have always focused on identifying the psychological factors contributing to the occurrence or severity of accidents.^19^ Most previous studies have examined the effect of psychiatric disorders on driving performance.^10,13,14,17^ Given that no study was found on the impact of psychiatric disorders on pedestrians’ traffic behavior, the present study was aimed to investigate the role of psychiatric disorders in pedestrians’ traffic behavior in Tabriz, Iran.

## Materials and Methods

###  Study Design

 This study is a cohort-based cross-sectional study which was carried out in 2022 in Tabriz. The data that were evaluated in the present study belonged to the pedestrians who participated in the Persian Traffic Cohort (PTC) which is the Iranian population cohort for road safety (Ethical Code: IR.TBZMED.REC.1395.1138).^20^

###  Participants

 The target population of the PTC involved the inhabitants of Tabriz, and the reference population involved the inhabitants of the two municipal districts of Tabriz. Cluster sampling was utilized to select the study sample. For this purpose, the neighborhoods of Tabriz were divided into three strata, containing low, medium, and high levels of socioeconomic status (SES). The list of households and the household members of the two municipal districts of Tabriz was firstly taken from the health center database of Tabriz. Then, 20 neighborhoods were randomly selected from all strata as clusters. The number of clusters in each stratum was determined according to the proportion of neighbors in each stratum to the total neighbors. Next, within each cluster, 24 households were randomly chosen. At the end, all individuals in each household entered the study. Out of 24 households who filled the primary questionnaires, about four households did not accept to refer to the PTC center for physician and psychiatric examinations and other tests. Therefore, the participation rate in the current study was about 83%. The individuals who referred to the PTC center were evaluated in various aspects consisting of field assessments via self-reported questionnaires, physical examinations, psychiatric examinations, para-clinical assessments, and traffic laboratory assessments using driving simulator, Vienna Test System, etc.

 In the current study, random sampling was used to include individuals with completed psychiatric questionnaires and Pedestrian Behavior Questionnaire (PBQ). Therefore, a total of 275 individuals of PTC who had the eligibility criteria entered the study (90% of available data). Inclusion criteria were as follows: samples who (1) were aged above 14 years, (2) lived in Tabriz second district municipality, 3) scored three or higher in the Structured Assessment of Personality Abbreviated Scale (SAPAS) questionnaire or scored 20 or higher in the Kessler questionnaire, (4) completed both Pedestrian behavior questionnaire and Structured Clinical Interview for DSM-IV *** (*** SCID DSM-IV), and (5) signed written informed consent. Exclusion criteria were: (1) unwillingness to continue the study, (2) incomplete filling of one of the mentioned questionnaires.

###  Data Gathering

 First, the Pedestrians Traffic Behavior Questionnaire, SAPAS, and Kessler’s screening questionnaires for people over 14 years of age were completed through door-to-door visits. Then, these people underwent a psychiatric interview (based on the SCID DSM-IV semi-structured interview) by a psychiatrist or psychiatric resident at the Traffic Accident Prevention Research Center.

###  Measurements

####  Pedestrian Behavior Questionnaire 

 The questionnaire consisted of two parts. The first part included demographic questions, and the second part consisted of 29 questions measuring pedestrian traffic behavior in five domains: (1) adherence to traffic rules (7 questions), (2) violation (10 questions), (3) positive behavior (6 questions), (4) distraction (4 questions) and (5) aggressive behavior (2 questions). The questions were scored on a five-point Likert scale (*never* = 5, *always*= 1). The dimensions of aggressive behavior, violation, and distraction were reverse-scored to align with questions of a positive nature (adherence to traffic rules and positive behavior). The minimum score was 29 and the maximum score was 145. In this study, the scores were converted to the 0‒100 range, and finally, the scores of different sub-scales were calculated. High scores in each dimension indicated safer pedestrian behaviors. Haghighi et al confirmed the validity of the questionnaire with the content validity index (CVI) of 87%, and its reliability with Cronbach’s alpha of 84%.^21^

####  Structured Assessment of Personality Abbreviated Scale 

 SAPAS is an eight-item questionnaire to diagnose personality disorder. Each item is scored 0 (No)/1 (Yes), except for question 3 which is inversely scored 1 (No)/0 (Yes). The personality score, calculated by summing each item’s reported scores, is a number between 0 and 8. Higher scores indicate the severity of the disease. A score above 3 on this questionnaire requires a visit to the psychiatrist.^22^ Sepehri et al found an area under the curve (AUC) of 0.566 (95% confidence intervals [CI]: 0.455‒0.677); sensitivity of 0.89 and specificity of 0.26 at the cut-off score of 2 and higher. The total Cronbach’s alpha coefficient was 0.38 and Cohen’s kappa ranged between 0.5 and 0.8 in their study.^23^

###  Kessler Psychological Distress Scale (K10)

 The Psychological Distress Scale was developed by Kessler et al to measure mental disorders. It has 10 items and measures psychological distress based on the four-point Likert scale (*not at all*= 1 to *always* = 5). The total score is obtained from the sum of the scores of the 10 items. Scores range from 10 to 50, and a higher score indicates greater severity of the disease. A score above 20 on this questionnaire requires a visit to the psychiatrist.^24^ In a study by Yaghubi, confirmatory factor analysis confirmed the K10 questionnaire single factor and the values of factor loadings for the main factor were between 65% and 84%. The sensitivity, specificity and overall misclassification rates for the best cut-off point of the Kessler Psychological Distress Scale (8) were 81%, 80.5% and 16.5%, respectively. Cronbach’s alpha reliability coefficient of the Scale was 93%.^25^

###  Structured Clinical Interview for DSM-IV

 The Structured Clinical Interview for DSM-IV (SCID DSM-IV) is a semi-structured interview to diagnose psychiatric disorders in adults according to the Diagnostic and Statistical Manual, fourth edition (DSM-IV). The semi-structured nature of the interview is because its administration requires the interviewer’s clinical judgment of the interviewee’s responses; therefore, the interviewer must have clinical knowledge and experience in psychopathology. The SCID is available in two versions: The Research Version and the Clinician Version. The study used the clinical form of SCID-IVA. This form has been used in psychiatric studies more than any other diagnostic interview and has universal credibility. Previous studies have confirmed the validity of the Persian version of SCID-IV for use in clinical and research settings.^26^ In a study by Mohammadkhani et al, SCID psychometric properties indicated an acceptable range for internal consistency (0.95‒0.99), test-retest reliability (0.60‒0.79), and kappa reliability (0.57‒0.72). Further, the agreement between interviewer and psychiatrist diagnoses obtained using κ statistics was good to excellent for all diagnostic categories (κ = 0.63 to κ = 0.83).^27^

###  Statistical Analysis

 The data were analyzed in SPSS v. 26. The normality of the data distribution was checked using the Kolmogorov-Smirnov test. Frequency (percentage) was used to describe qualitative data, and mean (SD) was used to describe quantitative data based on the normality of data distribution. Pedestrian traffic behavior was the dependent variable, and psychiatric disorders were the independent variables. Independent samples *t*-test, analysis of variance (ANOVA), and multiple regression analysis were used to examine the relationship between independent and dependent variables. The statistical significance level was < 5%.

## Results

 This study examined 275 pedestrians, mostly women (56.73%). The mean age of the participants was 42.45 (18.3) years. The majority were married (80.36%), and about 27% of the participants had a university education. More than half of the participants (68.36%) had no mental disorders. About 20% of the participants had generalized anxiety disorder, and almost 17% had major depressive disorder. Table 1 summarizes the information on the demographic characteristics of the participants.

**Table 1:** Demographic Characteristics of the Study Population (N = 275)

**Characteristics**	**No. (%)**
Age (y), means (SD)	42.45 (18.3)
Gender	
Male	118 (43.38)
Female	154 (56.62)
Marital status	
Single	54 (19.64)
Married	221 (80.36)
Educational level	
Non-academic education	201 (73.09)
Academic education	74 (26.91)
Number of psychiatric disorders	
0	188 (68.36)
1	48 (17.45)
2 or more	39 (14.18)
Diagnosed with GAD	
Yes	57 (20.73)
No	218 (79.27)
Diagnosed with MDD	
Yes	49 (17.82)
No	226 (82.18)

GAD, generalized anxiety disorder; MDD, major depressive disorder.

**Figure 1:**
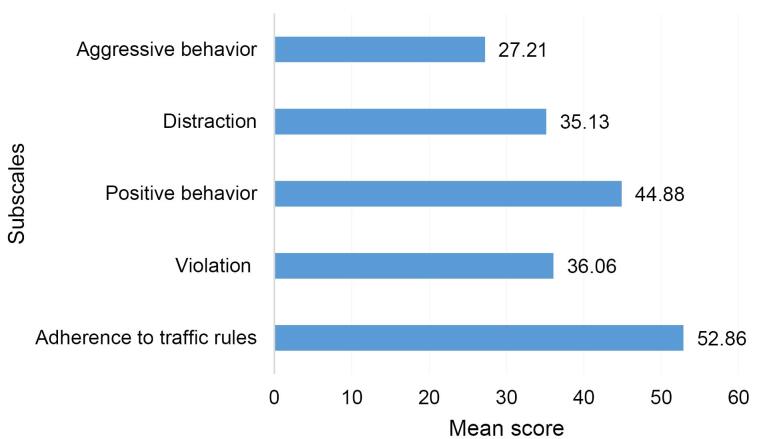
Normalized Mean Scores of Subscales of Pedestrian Behavior

Figure 1 displays the average standardized score of the dimensions of pedestrian traffic behavior. According to this figure, the lowest score belonged to the dimension of aggressive behavior (27.21). The mean total pedestrian traffic behavior score was 41.17.

 The adherence score to traffic rules was significantly higher in the pedestrians with more than one mental disorder than in the pedestrians with one mental disorder (*P *value = 0.005). However, the scores of violation, positive behaviors, distraction, aggressive behaviors, and the total traffic behavior score did not significantly differ across the three groups of pedestrians (no disorder, one disorder, and more than one disorder) (*P *value > 0.05) (Table 2).

**Table 2:** Relationship between Status of Psychiatric Disorders and Traffic Behavior among Pedestrians

**Pedestrian Behavior and its Subscales**	**Status of Psychiatric Disorders**	Mean Scores^*^ ** (SD)**	**95% CI**	***P *** Value^**^	**F**
Adherence to traffic rules	Without psychiatric disorder	52.12 (14.21)	49.99 – 54.24	5.37	0.005
	One psychiatric disorder	49.96 (18.09)	44.39 – 55.53		
	Two or more psychiatric disorders	60.60 (15.13)	55.24 – 65.97		
Violation	Without psychiatric disorder	34.96 (10.08)	33.41 – 36.53	3.43	0.340
	One psychiatric disorder	36.86 (9.67)	33.92 – 39.81		
	Two or more psychiatric disorders	40.29 (15.91)	34.74 – 45.84		
Positive behavior	Without psychiatric disorder	44.47 (16.12)	41.96 – 46.98	0.17	0.844
	One psychiatric disorder	45.96 (17.61)	40.54 – 51.39		
	Two or more psychiatric disorders	45.52 (16.21)	39.67 – 51.36		
Distraction	Without psychiatric disorder	35.11 (16.85)	32.59 – 37.63	1.06	0.349
	One psychiatric disorder	37.5 (17.07)	32.54 – 42.25		
	Two or more psychiatric disorders	32.16 (16.05)	26.81 – 37.51		
Aggressive behavior	Without psychiatric disorder	26.05 (14.01)	23.99 – 28.11	1.70	0.185
	One psychiatric disorder	29.37 (16.55)	24.56 – 34.18		
	Two or more psychiatric disorders	30 (17.24)	24.33 – 35.66		
Total PBQ score	Without psychiatric disorder	40.33 (7.92)	38.96 – 41.69	2.46	0.088
	One psychiatric disorder	41.83 (9.25)	38.56 – 49.16		
	Two or more psychiatric disorders	44.36 (12.13)	39.56 – 49.16		

PBQ, Pedestrian Behavior Questionnaire. * The possible range of subscales and total score of PBQ is 0 to 100. **P value by one-way ANOVA.

 There was no statistically significant difference between the two groups of pedestrians with and without generalized anxiety disorder in the scores of traffic behavior dimensions (adherence to traffic rules, violation, positive behaviors, distractions, aggressive behaviors) or the total score of traffic behavior (*P* value > 0.05) (Table 3).

**Table 3:** Relationship between GAD and Traffic Behavior among Pedestrians

**Pedestrian Behavior and its Subscales**	**Diagnosed with GAD**	Mean Scores* (SD)	**95% CI**	**t**	***P *** Value^**^
Adherence to traffic rules	No	52.28 (15.04)	50.19 – 54.36	-1.22	0.221
	Yes	55.27 (16.52)	50.53 – 60.02		
Violation	No	35.32 (9.98)	33.89 – 36.75	-2.06	0.461
	Yes	38.82 (14.38)	34.77 – 42.87		
Positive behavior	No	45.39 (16.07)	43.07 – 47.70	0.93	0.351
	Yes	42.91 (17.44)	37.85 – 47.98		
Distraction	No	35.34 (17.04)	85.3 – 92.4	0.38	0.791
	Yes	34.35 (16.07)	29.96 – 38.74		
Aggressive behavior	No	26.49 (14.54)	24.52 – 28.46	-1.54	0.123
	Yes	30 (16.55)	25.52 – 34.47		
Total PBQ score	No	40.81 (8.18)	39.51 – 42.09	-1.12	0.262
	Yes	42.54 (11.26)	39.03 – 46.05		

CI, confidence interval; PBQ, Pedestrian Behavior Questionnaire; GAD, generalized anxiety disorder. * The possible range of subscales and total score of PBQ is 0 to 100. **P value by independent samples t-test.

 The score of positive behaviors was significantly higher in the pedestrians group without depressive disorder than in the pedestrians with depressive disorder (*P *value < 0.001). Moreover, the score of aggressive behavior in pedestrians with depressive disorders was significantly higher than in pedestrians without depressive disorders (*P *value = 0.001). Nevertheless, the scores of other dimensions (adherence to traffic rules, violations, distractions) and the total score of traffic behavior did not differ significantly in the two groups with and without depressive disorder (*P *value > 0.05) (Table 4).

**Table 4:** Relationship between MDD and Traffic Behavior among Pedestrians

**Pedestrian Behavior and its Subscales**	**Diagnosed with MDD**	Mean Scores* (SD)	**95% CI**	**t**	***P *** Value^**^
Adherence to traffic rules	No	(15.01)52.28	50.19 – 54.36	-1.22	0.221
	Yes	(16.52)55.27	50.53 – 60.02		
Violation	No	(11.26)35.91	34.33 – 37.46	-0.51	0.604
	Yes	(10.51)36.90	33.54 – 40.26		
Positive behavior	No	(13.56)46.83	42.49 – 51.17	-0.82	**<0.001**
	Yes	(16.87)44.48	42.11 – 46.86		
Distraction	No	(16.99)35.26	32.96 – 37.55	0.25	0.801
	Yes	(15.97)34.57	29.88 – 39.26		
Aggressive behavior	No	(14.56) 26.92	24.98 – 28.87	-0.67	**<0.001**
	Yes	(17.09)28.54	23.60 – 33.48		
Total PBQ score	No	(8.98)40.89	39.51 – 42.28	-0.94	0.343
	Yes	(8.63)42.49	39.48 – 45.51		

CI, confidence interval; PBQ, Pedestrian Behavior Questionnaire; MDD, major depressive disorder. * The possible range of subscales and total score of PBQ is 0 to 100. **P value by independent samples t-test.

Table 5 reports the association of PBQ score with major depressive disorder (MDD), generalized anxiety disorder (GAD), status of psychiatric disorders, sex, age, educational level, and marital status. The results of multiple linear regression analyses showed that the PBQ score of pedestrians with MDD was 3.39 points higher than pedestrians without MDD (*P* = 0.226). The score of PBQ of pedestrians with GAD was significantly 6.64 points higher than pedestrians without GAD (*P* = 0.076). Being single compared to being married increased the score of PBQ by 1.27 (*P* = 0.548). Having non-academic compared to academic education increased the score of PBQ by 2.95 points (*P* = 0.059). PBQ score of male pedestrians was 2.69 points higher than female pedestrians (*P* = 0.060). No significant relationship was found between predictors and PBQ score (Table 5).

**Table 5:** Multiple Linear Regression Model of the Association between PBQ Score and Predictors

**Variables**	**Regression Coefficient**	**95% CI**		***P*** ** Value**
		**Lower Limit**	**Upper Limit**	
**Model 1:** Pedestrian behavior predicted by MDD, GAD, Status of psychiatric disorders, sex, age, educational level, and marital status (F = 2.27, *P* = 0.024, adjusted R-squared = 0.049, RMSE = 8.64)				
Diagnosed with MDD (No)				
Yes	3.39	-2.11	8.91	0.226
Diagnosed with GAD (No)				
Yes	6.64	-0.71	14.07	0.076
Status of psychiatric disorders (Without psychiatric disorder)				
One psychiatric disorder	6.49	-0.45	12.53	0.351
Two or more psychiatric disorders	13.79	-3.38	24.20	0.110
Sex (Female)				
Male	2.69	-0.01	5.49	0.060
Marital status (Married)				
Single	1.26	-0.89	5.43	0.548
Educational level (Non-academic education)				
Academic education	2.95	-0.01	5.89	0.059

CI, confidence interval;PBQ, Pedestrian Behavior Questionnaire; MDD, major depressive disorder; GAD, generalized anxiety disorder; RMSE, root mean square error.

## Discussion

 The study aimed to investigate the role of psychiatric disorders in pedestrians’ traffic behavior. The authors’ extensive search yielded no study on the relationship between mental health and pedestrians’ traffic behavior. Therefore, the results of the current study are discussed in terms of findings related to drivers.

 The present study indicated that among the different dimensions of pedestrian traffic behavior, the score of positive behaviors in pedestrians without depressive disorder was significantly higher than pedestrians with depressive disorder. Hopelessness is higher in those with depressive disorder, and they may even wish to die; therefore, less positive traffic behaviors in pedestrians with depressive disorder than those without depressive disorder seems psychiatrically justified. Moreover, the score of aggressive behaviors in pedestrians with depressive disorders was significantly higher than pedestrians without depressive disorders. This finding is also justifiable psychiatrically. Depression reduces concentration and prevents people from enjoying life; thus, depressed people have a lower tolerance threshold and, therefore, become more irritable and aggressive. Consequently, more aggressive traffic behaviors in pedestrians with depressive disorder than people without depressive disorder seem to be a reasonable finding.

 In the study by Elander et al, depression was associated with accidents leading to injury.^16^ Shinar et al also reported a significant correlation between neuroses, such as anxiety and depression, and road accidents.^28^ In the study by Soufi et al, carried out descriptively-analytically and retrospectively among motorcyclists, the traffic error score was higher in motorcyclists with major depressive disorder than those with mental health.^17^ In a cross-sectional study by Alavi et al, 800 Class A drivers were examined. The results showed that there was a significant difference between the average depression scores of drivers with a history of accidents and drivers without a history of accidents, and depression and anxiety increased the probability of car accidents by 3.6 and 2.6, respectively.^15^

 In terms of positive and aggressive behaviors, the findings of the present study are consistent with all the cited studies, indicating a significant association between depressive disorder and traffic behavior. However, the present study was inconsistent with all of the cited studies in terms of other variables that did not show any significant relationship with traffic behaviors. There are several reasons why the findings of this study are contrary to those of the mentioned studies, including differences in the target population and research settings in this study (pedestrians). This difference may also be due to differences in the sample size.

 In the present study, there was no statistically significant difference in terms of adherence to traffic rules, violation, positive behavior, distraction, aggression, and overall traffic behavior scores between the two groups of pedestrians with and without a generalized anxiety disorder. In line with the results of the present study, there was no significant correlation between drivers’ anxiety disorder and driving behavior in the study by Rahmani et al.^29^ Lack of a significant association between anxiety disorder and traffic behavior can be because people with anxiety disorder are constantly worried about unpleasant events, and they may often take precautions to avoid such events and adhere to standards more often. Therefore, the result is similar to that of people without anxiety.

 Alavi et al examined 800 bus and truck drivers prospectively. The findings showed that anxiety can increase the odds ratio (OR) of road accidents by 2.7 times.^13^ The results of the study by Fathi among Tabriz-Ahar Road drivers showed that the relationship between the components of physical symptoms, anxiety, social function, depression, and high-risk driving behaviors was significant.^30^ Asghari et al examined the relationship between anxiety, aggression, and driving behavior and showed that there is a negative correlation between anxiety and driving behavior; that is, anxiety and aggression had a negative effect on traffic behavior.^31^ According to the results of the relationship between anxiety disorder and traffic behavior, the present study was inconsistent with the studies of Alavi et al,^13^ Fathi,^30^ and Asghari et al,^31^ indicating a significant relationship between anxiety disorder and traffic behavior. Differences in the target population, research settings, and sample size can explain the difference in the results.

## Limitations and Recommendations

 Most of the previous studies have examined the role of psychiatric disorders in drivers’ traffic behavior. One strength of the present study is addressing the role of psychiatric disorders in pedestrians. The limitations of this study include a small sample size which may affect the significance of the results. Therefore, conducting studies with a larger sample size is recommended to increase the generalizability of the findings. Moreover, not all mental disorders were examined in this study. More comprehensive studies should be conducted, taking into account more mental disorders and larger samples.

## Conclusion

 In this study, only the adherence score to traffic rules dimension was significantly higher in those without a mental disorder and those with more than one mental disorder compared to pedestrians with one mental disorder. The two groups of pedestrians with and without generalized anxiety disorder did not have statistically significant differences in terms of adherence to traffic rules, violation, positive behavior, distraction, aggressive behavior, and the total traffic behavior score. Among the dimensions of traffic behavior, the score of positive behaviors was significantly higher in the pedestrians without depressive disorder than those with depressive disorder. Moreover, the score of aggressive behaviors in pedestrians with a depressive disorder was significantly higher than those without a depressive disorder.
